# A review of the biomechanical determinants of rugby scrummaging performance

**DOI:** 10.17159/2078-516X/2019/v31i1a7521

**Published:** 2019-01-01

**Authors:** A Green, Y Coopoo, J Tee, W McKinon

**Affiliations:** 1 Department of Sport and Movement Studies, Faculty of Health Sciences, University of Johannesburg, South Africa; 2 Movement Physiology Laboratory, School of Physiology, Faculty of Health Sciences, University of the Witwatersrand, South Africa; 3 Department of Sport Studies, Faculty of Applied Sciences, Durban University of Technology, South Africa; 4 Carnegie Applied Rugby Research (CARR) Centre, Institute for Sport, Physical Activity and Leisure, Carnegie School of Sport, Leeds Beckett University, Leeds, United Kingdom

**Keywords:** scrummage, force, kinematics, kinetics, muscular strength

## Abstract

**Background:**

The scrum is a physical contest unique to the game of rugby union, important for determining match outcomes.

**Objective:**

This review will describe the current understanding of the kinetic and kinematic determinants of successful scrum performance to support coaching interventions and inform on future research.

**Methods:**

Literature review.

**Results:**

Individual and combined scrumming forces increase with playing level but there is no concurrent increase in body mass or player strength. There is very little variation in individual kinematics between individuals and across levels of play, suggesting that there are limited possible techniques for successful scrummaging. Live scrum contests are dynamic and require constant adjustments to body positions in response to increased compressive force and exaggerated lateral and vertical force components. Skilled performers are able to exert high levels of horizontal force while maintaining effective body positions within this dynamic environment.

**Conclusion:**

Success in scrummaging depends on the optimisation of joint angles and force production at the individual level, and the coordination of effort at a team level. The analysis presented here demonstrates that producing large scrum-specific forces and achieving the optimal ‘body shape’ are essential for successful scrum performance.

In rugby union, a scrum is awarded when a team knocks-on the ball, or to restart play in situations where the ball has become unplayable.^[1]^ A scrum is a contest between eight players (forwards) from opposing sides who are bound together and push in a coordinated strength contest for possession of the ball. Scrums are composed of eight players from opposing sides interlocked in a distinctive formation. Players are arranged into three rows: front row (loose-head prop, hooker and tight-head prop); second row (two locks); and the back row (two flanks and an eighth man). Props bind to the hooker by gripping tightly onto the waistband, while the hooker will clasp the props around the shoulders by gripping onto the jersey below the shoulder blades. The second row links together shoulder to shoulder binding to the front row by lodging their heads into the gap between the hips of adjoining prop and hooker. Back row players will bind onto the second row players. Specifically, flanks will attach themselves to the locks and place a shoulder to push onto the prop on their side of the scrum (either loose- or tight-head). The eighthman will bind between the hips of the second row players and maintain a forward push. A scrum will commence when the team has assumed their formation and front row players from the opposing sides interlock in a forceful, yet controlled manner. Typical scrum durations are approximately 3 ± 1.4 seconds^[2]^, with 20–30 scrums occurring per game.^[2–4]^

Effective scrummaging is a key determinant of team performance. Scrum dominance provides a platform for launching attacking play and allows for the disruption of an opponent’s attacking play. As a result of safety concerns, scrumming is highly regulated^[5]^ and frequently penalised by referees.^[6]^ Teams with dominant scrums are awarded more scrum-related penalties^[7]^, allowing them the opportunity to score points and gain territory.

Historically, scrum involvement resulted in a number of catastrophic neck and spinal injuries.^[5,8]^ In response, World Rugby has made changes to the rules governing scrums and particularly, the methods of front row engagement.^[9,10]^ While these changes have been effective,^[8]^ scrum laws continue to evolve. Coaches need to have a clear understanding of the determinants of effective scrumming to allow them to coach effectually and adapt these appropriately in response to the frequent rule changes.

The purpose of this brief review is to identify, explain and expand on the literature focussing on scrummaging force generation in order to illustrate the current scientific understanding of scrummaging performance. The intention of this review is to better isolate key performance factors which may facilitate future research and produce more successful yet safer scrummaging performance training programmes.

## Kinetics

Scrum kinetics have been used as the major objective scientific measure of scrummaging performance. Various methods have been employed to quantify this which include: instrumented scrum machines^[11–16]^, force platforms^[17]^, and shoulder mounted pressure transducers.^[18–20]^

### Force components

The force exerted in the scrum is composed of compressive, lateral and vertical forces.^[10,16,21,22]^ The lateral forces have been found to be directed towards the tighthead prop (right)^[10]^ and attributed to the wheeling of the scrum.^[16,20]^ Vertical or shear force has, in turn, been associated with scrum collapses and front row players coming out of formation.^[10,19,20]^ Even though lateral and vertical forces contribute to scrum contest outcomes, the compressive force (i.e. forward pushing force) is of most interest to investigators and coaches due to its obvious performance implications. The compressive, vertical and lateral forces present during scrumming are the result of the kinetic capabilities of the team’s scrum as a unit, which are comprised of the distinct individual kinetic capabilities of each player. However, combined pack kinetics do not equal the sum of individual kinetics due to the compression of soft tissue and the cancellation of interactions between players within the scrum pack.^[14,15]^

### Individual force contributions

Scrum contests are usually won by packs with larger combined compressive forces.^[23]^ Assessments of team scrummaging have identified the contribution of various playing positions in terms of the total scrummaging forces. Interestingly, the front rows contribute the most force, namely between 42% – 46%, and locks generate between 21% – 37%, respectively. On the other hand, the loose forwards contribute the least of between 21% and 30% of the total scrummaging force.^[14,24]^

Although the different playing positions in the scrum contribute varying magnitudes of force, they may tend to use the directional components of these forces to varying degrees. For example, in addition to the 21% – 30% contribution to total scrum force, loose forwards also assist the tight five players by improving scrum stability.^[14,16,20]^ du Toit et al.^[14]^ showed that the largest lateral force application angles were produced by tight-head flankers. Therefore, flankers act as a wedge which assists in developing larger compressive forces and maintaining the forward direction of their props, which may be displaced by the second row’s (locks) force application angles.^[14,25]^

Force magnitudes measured from individual studies vary greatly (Table 1). Variations are due to measurements at different points in time, levels of playing proficiency of the study’s participants (both in terms of playing position and level of competition) and surfaces. Despite this, the individual peak force of scrummaging may exceed 3000 N in idealised indoor settings, yet it is slightly lower than 2500 N on natural turf (although the latter have only been assessed in sub-elite players). Peak forces may be a result of the engagement force, which is significantly larger than the sustained force.^[10,18,21,22]^ Thus a peak force may not truly reflect an individual’s ability to exert a similar magnitude during the sustain phase of the game.

New scrum laws have had a considerable effect on scrummaging kinetics where bind and set phases attempt to make the engagement safer by reducing the collision between the front rows.^[6,9]^ Additionally, the new laws prevent teams from pushing before the ball is fed into the scrum. However, from a kinetic perspective, this procedure complicates the contest since an initial low-level contest is introduced prior to the dynamic one. This means that the scrum must remain steady and the packs must exert a certain level of force to keep the scrum stationary. Once the ball has been fed into the scrum, teams can actively compete for the ball, which should result in a second force peak. Therefore, a team that can sustain a larger force magnitude during the steady state and actively generate a ‘second shove’ once the ball is fed, may achieve better scrum outcomes than the previous isolated engagement or sustained forces under the older rules.

There are numerous difficulties comparing combined pack scrummaging forces across multiple studies. The first issue is the change in scrummaging rules, which have reduced the engagement force.^[18]^ However, data presented by Preatoni et al.^[10]^, and Cazzola et al.^[19]^ illustrate that the sustained compressive force remains unchanged regardless of the engagement procedure. Therefore, Table 2 reports the sustained compressive forces for pack scrummaging. A second concern is the devices used to measure the compressive force. Most studies have used static instrumented scrum machines; however, du Toit et al.^[20]^ and Cazzola et al.^[19]^ used shoulder mounted pressure sensitive pads during live scrums. Based on these various collection methods, a large range of force values are reported in this review. Specifically, the large discrepancies between data reported by Preatoni et al.^[10]^ and Cazzola et al.^[19]^ may be indicative of the methodologies employed. Static instrumented scrum machines are less likely to overestimate forces due to their rigidity. However, while shoulder mounted force sensors may underestimate force magnitudes due to tissue artefacts between the opposing front rows, they give a better description of live scrum contests.^[18]^

Front row binding involves the interlocking of two rows of three players each where their heads will be positioned between two opposing players. Due to the binding offset, loose-head props will only have one contact point (the shoulder of their opposing tight head prop) which allows a greater range of motion and the possibility of generating larger lateral and vertical forces. Additionally, front rows experience a larger force on their shoulders when scrummaging as a pack compared to individually, which can be attributed to the summation of force from the locks and loose forwards respectively.^[20]^

Despite variations in rules and force measurement techniques, scrummaging force magnitude has been found to increase with the playing level^[10,33]^, which may result from increasing player mass and strength. However, no correlation between either body mass or strength measures and maximal horizontal scrummaging forces in professional players exists.^[17]^ Players of similar body mass and strength must therefore be using different scrummaging techniques to achieve their maximal scrummaging forces.^[10,17]^ These technical parameters may be based on movement (kinematic) strategies^[10,34]^ or achieved through the coordination of exerted forces within the scrum.^[20]^

## Kinematics

Features of an ideal scrum position were introduced by Milburn^[24]^ who suggested that the head, including the neck, trunk and legs all be aligned parallel to the direction of the intended force. Additionally, it was suggested that a greater angle (sagittal plane view) between the trunk and legs (hip angle) results in a larger force.^[24]^ Most studies have, however, been descriptive in nature. The following section summarises the findings of these studies, with a kinematic description of the scrum sequence spanning the preparation, engagement, steady state (pre-ball feed) and contest (post-ball feed) phases.

### Preparation phase

The scrum engagement sequence begins with the players in a crouched position. During the preparation phase, prior to the two front rows engaging, players bind to their opponents by gripping onto their jerseys. Front row players are instructed to have their shoulders above their hips (when viewed in the sagittal plane) to prevent the scrums from collapsing resulting in their trunks being slightly above the parallel relative to the ground.^[19,24,34]^

In this preparation phase, the players have their feet firmly on the ground with a large degree of flexion at the hips and knees. Wider foot stances may influence the generation of scrummaging forces.^[30]^ Foot orientation may be slightly everted to allow for a larger ground contact area relative to the direction of the imparted force. Most forwards will adopt a parallel foot stance on set-up, prior to the scrum contest; however, a minor offset between the feet may be present.^[13,32]^ Of importance is that Sayers^[34]^ showed while starting positions may differ, body positions upon engagement are similar. Therefore, the preparation phase of scrummaging may only be a result of player preference and their ability to maintain balance prior to scrum engagement.^[17]^

### Engagement phase

On the call of “set”, front row players rapidly extend their hips and knees^[34]^ and in a controlled manner and make impact with their opponents. It is during the engagement phase that the generation of maximal compression force is usually exerted.^[19,21]^ Combined vertical force components are initially directed downwards but continually shifts upwards as scrummaging duration continues.^[10]^ The kinetics of the scrum therefore, closely represent the kinematic changes which occur.^[30]^

Du Toit et al.^[14]^ stated that the front row requires vertical stability before being able to apply force. Furthermore, du Toit et al.^[14]^ suggested that the front row make a deliberate effort to scrum higher up to prevent the scrum from potentially collapsing. It can be presumed that starting at a lower, more flexed position could be beneficial as the player could produce greater upward force through the extension of their hips and knees.^[30]^

The sustained force phase follows the engagement phase.^[10]^ Forces during the sustained phase fluctuate around a constant magnitude which is lower than the force produced during the engagement phase.^[10]^ Of importance is that the sustained force phase as measured on scrummaging ergometers may not reflect the dynamic nature of a live scrum contest. In line with the most recent definitions of the law, the sustained phase is divided into steady-state (during which force magnitudes are maintained) and the contest phase (once the ball enters the scrum tunnel and players are required to strike or contest for the ball). The latter phase is yet to be replicated on a scrum machine or kinematically evaluated during live scrummaging.

### The steady state phase (pre-ball feed)

The steady state phase, which occurs on immovable scrummaging machines, reflects the sustained force period with the players remaining in a largely isometric position. During the sustained phase, the lower limbs exhibit a large degree of extension^[13,15,30,31,34]^, and the trunk will gradually rise above the horizontal position.^[18,19]^ This movement and body position may result in players ‘overextending’ which could cause the scrum to collapse. Statistically significant relationships have been presented between the extensions of the hip (r=−0.47), knee (r=−0.51) and the ankle (r=−0.70) and the individual scrummaging forces.^[13]^ Bayne and Kat^[32]^ inferred correlations for ankle dorsiflexion (r=−0.12), trunk extension (r= 0.32) knee (r= −0.63) and hip flexions (r= −0.74) angles and the compression force. Other researchers have failed to show relationships between kinematics and scrummaging performance.^[15,30]^ Collectively, these findings do not provide conclusive relationships between force development and scrummaging body positions. Methodological differences, players’ skill levels and ecological constraints may further compound the difficulties in finding distinct movement patterns related to scrummaging force development. Table 3 collates joint angles from various individual scrummaging assessments at maximal sustained force. The similarities in the individual kinematics reported in this review suggest that there are limited techniques to scrummaging. Further evidence suggests that proficient players adopt a similar body position over the scrum’s duration^[34]^ with little axial skeleton movement variability.^[35]^ Thus it is possible that the body position optimal for force production is fundamentally safe and effective.^[8]^ Finally, an effective scrummaging position may require obtaining individualised optimal length-tension relationships in the primary muscles rather than attaining particular joint angles. Further research into the relative contributions of different muscles, muscle coordination and individualised force-tension relationships of major muscle groups to the overall force generation may deepen the understanding of muscle force production during scrummaging.

With regard to the effects of feet positioning adopted during the preparations phase, no significant difference in the exerted forces were reported irrespective of the feet’s positions.^[13]^ However, a double peak force pattern is exhibited in the cross-feet position, compared to the single peak in the parallel feet position. An offset foot stance could result in larger lateral forces on the side of the lead leg diminishing the total compressive force of the scrum^[32]^ and may cause the scrum to wheel. Additionally, these increased lateral forces may cause excessive spinal rotation experienced by the individual players, as the hip may act as a pivot around which the axial rotation of the trunk can occur.

### The contest phase (post-ball feed)

The findings above focussed on static body positions during individual player scrummaging. However, the scrum is dynamic and requires adjustments in body positions resulting from the scrum contest. Measuring kinetics during live scrum contests is difficult, as the motion is dynamic, and the scrum cannot realistically be contested against an immovable object. Similar to scrum machine kinetics, shoulder mounted force sensitive devices recorded significantly lower sustained forces during a live scrum in comparison to the live engagement.^[20]^ However, greater fluctuations in the force may exist. During a scrum contest, players attempt to step forward. This will produce surges in the compressive force and exaggerate the lateral and vertical force components.^[32]^ Further confounding the issue is when a player strikes for the ball as stipulated by the law, they will remove one foot from the ground. This action will cause a reduction in the force magnitude. Therefore, in order to maintain the opposing pack force, the scrum pack will have to increase their individual force contributions. This highlights another gap in the understanding of scrummaging performance.

### Scrum contest complications

Kinematic analysis of scrummaging poses numerous difficulties. From a data collection perspective, it is easier to collect scrum kinematics on individual players compared to an entire pack. One solution may be to use wearable inertial sensors^[36]^ or modern video technology that does not require surface markers. Kinematic analysis is limited by its predominant use of scrummaging force as a performance index. The analysis is further limited by testing against an immovable object where an individual player can only perform isometric exertions. Furthermore, contest phases cannot be emulated against an immovable ergometer. While this method is ultimately the gold standard for measuring scrummaging forces, more representative methods are required to measure pack power, forces and velocities. Despite these shortcomings, the measurement of technical variables identified through kinematic analysis may assist in training drills and aid in the development of good techniques.^[35]^ Relationships between the generation of scrummaging forces and specific body (or joint) positions may, however, be difficult to reveal. It is possible that the ability of the muscles to generate torque around these joints may provide additional insight into force generation and performance in the scrum.

## Electromyography

The generation of scrummaging force during the engagement sequence is a result of muscular contraction. The majority of muscles investigated in scrummaging studies are predominately those acting on the back, hips, knees and ankles.^[17,37–39]^ As scrummaging is a measure of strength, standardised amplitudes should be related to its performance. However, Sharp et al.^[17]^ reported no significant correlations between EMG activation levels and scrummaging forces. A possible reason for this lack of relationship could be as a result of the players adopting similar positions and reduced movement, and EMG variability during machine scrummaging.^[37,38]^ Additionally, stronger players may require less muscle activation to produce similar force.^[37]^

The activation patterns of muscle prior to and during the engagement sequence may reveal important muscular contributions to force generation. The following section briefly identifies maximal activations at specific time-points before the nature of muscle activity over the entire scrum effort duration is described.

Prior to scrum engagement, the gastrocnemius muscle is largely activated and the vastus lateralis reaches maximal activation, as the knees are rapidly extended.^[17,37]^ Back musculature, such as the erector spinae, are largely activated in the preparation phase.^[17,38,39]^ The large muscle activation of the erector spinae sequence can be attributed to the crouched position of the player prior to engagement.^[17,39]^ Cazzola et al.^[39]^ suggested that the muscles of the back and neck are primed prior to scrum engagement which could increase joint stiffness of the back and neck. Although the increased joint stiffness may adequately stabilise the trunk to assist in the transmission of forces, it may be insufficient to prevent injury. This premise is supported by the highly active erector spinae group during sustained force scrummaging.^[17,38,39]^

Assessment of the proximal muscles, particularly those of the back and the abdomen, reveal that the abdominal muscles are not significantly activated^[38]^ over the entire duration. Additionally, there is minimal activation of the biceps femoris over the entire scrummaging sequence compared to the rectus femoris and vastus lateralis respectively.^[17,38]^ More distally, the gastrocnemius experiences large activation patterns throughout the scrummaging sequence.^[38]^

An electromyographical assessment obtained during machine scrummaging is not representative of those obtained during live scrums, even though kinetics and kinematic parameters are closely related.^[38,39]^ The dynamic nature of live scrummaging requires more reactive muscle activity. Before being able to effectively apply force, the front row needs to establish stability in order to allow the forces generated by the rest of their pack to be effectively transmitted through the scrum onto their opponents. This is confirmed by large erector spinae muscle activity of front row players reported during live scrums.^[39]^

## Muscular strength and power

By definition, muscular strength is the ability to exert force on an external object^[40]^, and therefore strength is essential for scrum performance. Scrum forces and player strength increase at the higher levels of the game.^[41,42]^ Despite this, so far researchers have failed to demonstrate meaningful relationships between strength measures and scrum force production.

Logically, the largely isometric action of scrum contest suggests that multi-joint isometric strength measurements would be the best indicators of individual scrum force production. However, Quarrie and Wilson^[15]^ failed to show a relationship between scrum force and strength in a modified isometric mid-thigh pull. Similarly, no relationship has been demonstrated between vertical jump heights and scrummaging force production.^[15,30,43,44]^ However, Quarrie and Wilson^[15]^ reported weak relationships (r= 0.39–0.41) between individual scrum force and maximal isokinetic knee extension torque at two velocities, while Sharp et al.^[17]^ showed no difference in isokinetic tests across playing levels. Individual scrummaging force is a strength measure, but meaningful associations with other more traditional strength measures have yet to be clearly established.

### Combined pack mass, strength and power

On a population level, body mass and strength are closely related.^[45]^ Therefore, it is not surprising that researchers have shown significant relationships between scrum force and combined pack mass.^[10,13–16,20,24,30]^ In the only study to have considered a combined power metric, Green et al.^[23]^ demonstrated that winning scrums also had significantly higher combined vertical jump heights.

However, du Toit et al.^[20]^ reported that while a significant relationship exists between pack mass and combined engagement force, no relationship exists between sustained scrummaging force and pack mass. In this case, fat mass may be a confounder. The reason for this being that while additional fat mass may contribute to engagement momentum, it cannot assist in generating any sustained scrummaging force. Additionally, Preatoni et al.^[10]^ reported that increases in the compressive force magnitudes in various playing levels were not dependent on pack mass. Finally, Green et al.^[23]^ reported no association between combined pack mass and the outcome of numerous scrum contests. Therefore, it is likely that team scrum performance results from the force production, and technique and timing capabilities of the players rather than combined player mass.^[10,15,20,24]^ However, at an age-group level or non-elite level, a difference in mass may be the determining factor to scrum success.

### The role of fatigue

Scrum performance appears to be largely unaffected by the fatigue levels of the players. Scrum force production has been shown to be reduced after repeated scrum efforts interspersed by 20 second rest periods^[28]^ but was unchanged when the rest periods were increased to 30 seconds^[29]^, following a rugby-specific fatigue intervention^[31]^, and repeated sprint activity.^[28,46]^ Similarly, scrum kinematics were also unaffected by fatigue.^[31]^ Two explanations for this are that fatigue interventions employed in research have been insufficient to induce specific fatigue, or that rugby players develop the ability to maintain a competitive scrummaging force and body positioning under fatigue conditions ^[31]^.

### Scrum tactics: Exploiting technical performances

Despite the emphasis on scrum force and body position in this review, in game settings scrum outcomes are also affected by tactics. During scrum contests, players may employ coached techniques that reduce their opponent’s ability to scrum effectively. Ideally, front row players should contest directly against their opposite number, that is, the loose-head prop should push against the opposing tight-head prop. However, players frequently change height and angles at which they push to unsettle their opponent. As an example, loose-head props may attempt to “get underneath” their opposing tight-head prop - with the aim to push them up and in towards their hooker rather than directly backwards. While illegal, these subtle variations in scrummaging technique are notoriously difficult on which to make a formal judgement.^[7]^

Other tactics employed include the deliberate wheeling of the scrum^[14]^, facilitated by teams deliberately changing their foot position.^[32]^ Defending teams have also been known to wait for the attacking team to hook the ball (necessitating one player taking a foot off the ground), to produce a coordinated shove thereby taking advantage of this moment of instability. While it is likely that the scrum with the greater force production capacity will still dominate these contests, the skill required for players to maintain force production dynamically adjusting to this highly variable system should not be underestimated.

## Conclusion

The scrum contest is one of the quintessential parts of a rugby game. Success here depends on the optimisation of joint angles and force production at the individual level, and coordination of effort at a team level. Analysis presented here demonstrates that producing large scrum-specific forces and achieving the optimal ‘body shape’ are essential for scrum performance. Clear relationships between muscle activation, strength, fatigue and scrum performance have yet to be demonstrated. This is likely the result of studying a skill with limited available technique options in a largely homogeneous group of players.

Coaches should use scrum machines to teach individual kinematics, train scrum-specific strength and develop team coordination. Live scrum training induces variability in the task that it is essential that players learn to manage to be consistently successful in dynamic competitive scrums. Individual skill, inter-player timing and familiarity are likely to be factors that can positively relate to team scrummaging performance.

## Figures and Tables

**Table 1:** Individual scrummaging force magnitudes, playing levels and ground compositions from recent publications

Study	Individual force magnitude (N)	Body mass (kg)	Playing level	Measurement of maximal force	Ground composition
Quarrie and Wilson [15]	1370 ± 280	96.9 ± 9.8	Premier club	Peak	Synthetic matting
Hot et al. [26]	1466 ± 244	96.9 ± 10.1	Club elite	NS	NS
Wu et al. [13]	1171 ± 277*	85.5 ± 9.61	National	Peak	Indoor
Sharp et al. [17]	4493 ± 151	112.1 ± 6.5	Professional	Peak	Synthetic matting
Sharp et al. [17]	3091 ± 653	101.4 ± 9.3	Senior amateur	Peak	Synthetic matting
Sharp et al. [17]	3362 ± 788	99.1 ± 6.0	Junior amateur	Peak	Synthetic matting
Mensaert et al. [27]	3205 ± 3093	NS	Junior amateur	Peak	Indoor
Mensaert et al. [27]	3076 ± 1014	NS	Senior amateur	Peak	Indoor
Mensaert et al. [27]	5010 ± 1195	NS	Professional	Peak	Indoor
Cazzola et al. [19]	2800 ± NS	102.4 ± 15.0	University 1st XV	Peak	Indoor
Morel et al. [28]	1609 ± NS	90.9 ± 9.8	Elite u-23	Mean sustained over 5 seconds	Synthetic track
Green et al. [11]	2254 ± 649	101.0 ± 14.1	Club amateur/university	Peak	Natural turf
Morel and Hautier [29]	1741 ± 207	103.3 ± 11.8	Elite u-23	Peak during engagement phase	Artificial turf
Green et al. [23]	2274 ± 636	99.0 ± 18.2	Club amateur/university	Peak	Natural turf
Green et al. [30]	2458 ± 455	103.0 ± 12.1	Club amateur/university	Peak	Natural turf
Green et al. [31]	1720 ± 363	106.2 ± 13.3	University 1st XV	Peak	Indoor
Bayne and Kat [32]	2290 ± 410	100.7 ± 15.0	Club amateur/university	Mean sustained resultant force over 9.5 seconds	Natural turf Sprinting blocks

Data are expressed as mean ± SD

NS, not specified within text; N, Newton;

* calculated from percentage of average body mass and converted to force.

**Table 2:** Pack playing levels, weights and sustained compressive forces during team scrummaging

Study	Playing level	Pack weight (N)	Sustained compressive force (N)
Milburn [16]	High school	5588	3370
	University	6726	4160

Quarrie and Wilson [15]	Premier rugby	7570 ± 350	7234 ± 726*

du Toit et al. [14]	High school	NS	6848 ± 1140

du Toit et al. [20]	High school	6406 ± 235	6146 ± 1337

Preatoni et al. [10] (crouch touch set call)	School	6685 ± 637	4900 ± 1300
	Women	6326 ± 257	4800 ± 500
	Academy	7771 ± 197	5900 ± 800
	Community	8262 ± 325	5800 ± 400
	Elite	8523 ± 143	8000 ± 700
	International	8749 ± 165	8300 ± 1000

Cazzola et al. [19] (crouch touch set call)	Elite	8378 ± 275	3800 ± 1200

Cazzola et al. [19] (prebind)	Elite	8379 ± 275	3800 ± 1400

Data are expressed as mean ± SD

NS, not specified within text; N, Newton;

* Authors state that packs were able to exert 66% of the peak impact force during active scrummaging (sustained force)

**Table 3:** Kinematics of individual scrummaging attempts at maximal sustained force

Study	Sample size	Hip (°)	Knee (°)	Ankle (°)
Quarrie and Wilson [16]	56	123 ± 24	107 ± 13	78 ± 11
Wu et al. [13]*	10	121 ± 7	101 ± 18	62 ± 16
Mensaert et al. [27]	28	162 ± 73	101 ± 40	85 ± 25
Green et al. [30]	25	114 ± 17	144 ± 16	73 ± 16
Green et al. [31]	12	103 ± 33	124 ± 16	89 ± 18
Bayne and Kat [32]*	29	126 ± 17	129 ± 14	89 ± 7

Data are expressed as mean ± SD

* Feet in the parallel position.

Hip and knee angles have been adjusted to report the degree of extension (full extension denoted by 180°)
